# Network Pharmacology and Molecular Docking to Elucidate the Potential Mechanism of Ligusticum Chuanxiong Against Osteoarthritis

**DOI:** 10.3389/fphar.2022.854215

**Published:** 2022-04-14

**Authors:** Cheng Xiang, Yilin Liao, Zhuoyuan Chen, Bo Xiao, Ziyue Zhao, Aoyu Li, Yu Xia, Pingxiao Wang, Hui Li, Tao Xiao

**Affiliations:** ^1^ Department of Orthopaedics, The Second Xiangya Hospital, Central South University, Changsha, China; ^2^ Second Xiangya Hospital, Central South University, Changsha, China

**Keywords:** osteoarthritis, network pharmacology, molecular docking, MAPK pathway, ligusticum chuanxiong

## Abstract

**Background:** Osteoarthritis (OA) is a degenerative disease which serious affects patients. Ligusticum chuanxiong (CX) has been shown to have a certain curative effect on osteoarthritis in traditional Chinese medicine therapy. This study is based on network pharmacology and molecular docking technology to explore the potential mechanism of CX.

**Methods:** Components of CX to treat osteoarthritis were screened in the TCMSP database and targets were predicted by the PharmMapper database, the osteoarthritis targets were collected from the GeneCards database, and intersection genes were found to be the possible targets of CX anti-OA. The STRING database and Cytoscape software were utilized for protein-protein interaction analysis and further screening of core targets. The Metascape database was used for KEGG and GO enrichment analyses. Then, the top 10 pathways were selected to construct “drug-compound-target-pathway-disease” network analysis. Finally, molecular docking was used to analyze the binding affinity of seven compounds with core targets and TNF-α.

**Results:** Seven compounds with 253 non-repetitive targets of CX were screened from the TCMSP database and 60 potential intersection targets of CX anti-OA were found. PPI network analysis showed that the core targets were ALB, AKT1, IGF1, CASP3, MAPK1, ANXA5, and MAPK14, while GO and KEGG pathway enrichment analyses showed that the relevant biological processes involved in the treatment of osteoarthritis by CX might include the MAPK cascade and reactive oxygen species metabolic process. The KEGG pathway analysis result was mainly associated with the MAPK signaling pathway and PI3K-AKT signaling pathway. We further docked seven ingredients with MAPK1 and MAPK14 enriched in the MAPK pathway, and TNF-α as the typical inflammatory cytokine. The results also showed good binding affinity, especially FA, which may be the most important component of CX anti-OA.

**Conclusion:** Our research revealed the potential mechanism of CX in the treatment of OA, and our findings can also pave the way for subsequent basic experimental verification and a new research direction.

## Introduction

Osteoarthritis (OA) is a degenerative disease of the joints, it mainly occurs in knee joints and the clinical manifestations are pain, swelling, and limited movement (Katz et al., 2021). The pathological changes are cartilage degeneration, subchondral bone sclerosis, and synovitis (Abramoff and Caldera, 2020). It was reported that there are more than 250 million people around the world suffering from osteoarthritis, and half of the world’s population aged 65 and older suffer from OA (Litwic et al., 2013). Without radical treatment, osteoarthritis has become the main reason for the worldwide public problem of limb disability. Currently, non-steroidal anti-inflammatory drugs and intra-articular local injection of glucocorticoids were used as the first-line therapy for OA, mainly leading to reduced symptoms and restored joint function (Sharma, 2021). These drugs with short term efficacy not only cause serious damage to digestive tract, liver, and kidney function, but also accelerate the progress of arthritis in the long run (Dragos et al., 2017).

The research of traditional Chinese medicine (TCM) on osteoarthritis can be traced back to thousands of years ago (Yuan et al., 2016). It was first mentioned in “Huangdi Neijing”. Osteoarthritis is called bone arthralgia in TCM. According to the principle of diagnosis and treatment based on an overall analysis of the illness and the patient’s condition, the treatment for OA in TCM mainly focused on tonifying the liver and kidney, expelling wind and dampness, promoting blood circulation, and dispersing cold. Rich theoretical knowledge and therapeutic drugs have been accumulated for thousands of years (Wang C. et al., 2020).

The medicinal value of Ligusticum chuanxiong (CX) is in its roots and stems. It has been used in traditional Chinese medicine for thousands of years which was first mentioned in “Shennong’s herbal classic” (Chen Z. et al., 2018). It is one of the most important and commonly used drugs for promoting blood circulation and removing blood stasis. It is known as an “important medicine for headaches in various meridians” (Ran et al., 2011). It also plays a significant role in ischemic diseases, dizziness, and irregular menstruation (Wang M. et al., 2020). In addition, it also has a certain effect on rheumatism and joint pain, including osteoarthritis (Zhou et al., 2019a). Many researchers have focused on CX in recent years. In 1977, Chinese researchers first isolated tetramethylpyrazine (TMP), which is the most important alkaloid in CX (Sue et al., 2009). And 189 active components have currently been found, with some basic studies showing that the active components of CX can improve the vitality of chondrocytes, inhibit the apoptosis of chondrocytes, and upregulate the expression of type II collagen which is a major part of cartilage tissue (Ju et al., 2010). Undoubtedly, CX or its isolate compounds are potential candidates of the treatment for osteoarthritis in the view of modern drug development. However, only a few studies about CX have focused on the treatment of osteoarthritis, and the scope of these studies is not adequate (Ye et al., 2011; Luo Y. et al., 2020). Therefore, a new way or concept may be needed to help us sufficiently explore the mechanism of CX anti-OA.

CX, as with other traditional Chinese medicines, has complex action mechanisms and multi-channel interaction (Tao et al., 2013). In the past, traditional Chinese medicine was regarded as empirical medicine because of the lack of modern research methods and technologies (Luo E. et al., 2020). Network pharmacology is a new method of combining computer science and medicine which was first proposed by Hopkins in 2008 (Hopkins, 2008). Its interactive network based on “multi-gene”, “multi-target”, and “multi-channel” interactions coincides with the thinking and methods of TCM. Network pharmacology has become an emerging discipline to help us deeply study traditional Chinese medicine.

Active components of CX were screened and predicted by network pharmacology. By constructing the “drug-component-target-pathway-disease” interactive network and utilizing GO and KEGG pathway enrichment analysis and molecular docking technology, the potential molecular mechanism of CX in the treatment of osteoarthritis was explored. Our research may provide guidance for subsequent basic experimental research.

## Methods

### Pharmacokinetics and Component Screening of CX

The TCMSP database (http://lsp.nwu.edu.cn/tcmsp.php) (Ru et al., 2014) was utilized which can predict absorption, distribution metabolism, and excretion (ADME) characteristic information, such as oral bioavailability (OB), drug similarity (DL), Caco-2 permeability (Caco-2), and blood-brain barrier (BBB) issues. OB value is the most important feature of oral drugs, which can evaluate the efficacy of drugs distributed to the whole-body circulation after absorption. It is difficult to reach the effective concentration of most TCM components to exert their efficacy in specific tissues and organs, probably due to their small OB value. According to the recommendations of the TCMSP database and previous studies, OB value ≥30% is considered to have relatively reliable pharmacological activity. DL is a concept of physicochemical properties and molecular structure. It is based on the chemical structure of existing drugs to evaluate whether new compounds meet the characteristics of drugs, so as to become a new drug. The threshold DL value is 0.18 according to previous research (Zhang et al., 2020). We used the chemical name “Ligusticum chuanxiong” to search for its pharmacokinetic characteristics, and the active ingredients were further screened by taking oral bioavailability (OB) ≥ 30% and drug likeness (DL) ≥ 0.18 as the screening conditions (Ai et al., 2020).

### Target Prediction of CX Against OA

The reverse pharmacophore localization database PharmMapper (http://www.lilab-ecust.cn/pharmmapper/) (Liu et al., 2010) was used to predict the potential targets of CX. After screening the active components from the TCMSP database, the SDF structure formats of these small molecular compounds were obtained from the PubChem database (https://pubchem.ncbi.nlm.nih.gov/) and uploaded to the PharmMapper server. Species “*Homo sapiens*” was selected in uniport (https://www.uniprot.org/) (Uszkoreit et al., 2021) to standardize the Uniprot ID to the gene symbol. At the same time, the targets of OA were predicted in the Gene Cards database (https://www.genecards.org/). The keyword “osteoarthritis” was used to collect potential genes. We selected a correlation score >1 of OA targets, norm fit >0.6 of CX component targets for further analysis, and the overlapping area of the Venn diagram represented the potential targets of CX anti-OA.

### PPI Network Construction

The STRING (http://string-db.org; Version 11.5) (Feng et al., 2019) database was used to construct a protein-protein interaction (PPI) network for CX anti-OA to analyze the functional interaction between proteins. The network confidence score ≥0.4 was set to obtain targets with “*Homo sapiens*” being selected in Cytoscape software (version 3.8.1) further. The MCODE (molecular complex detection) and Cytohubba plug-ins were used to collect the core targets of CX anti-OA to construct the regulatory network.

### GO and KEGG Pathway Enrichment Analysis

GO function and KEGG pathway enrichment analyses were conducted to explore the core mechanism and pathway of CX anti-OA in the Metascape (http://www.metascape.org/) (Zhou et al., 2019b) database. We searched the gene symbols of common targets in Metascape by limiting the species to “*Homo sapiens*”, and setting the cut-off *p* value as 0.01 and min overlap as three for enrichment analysis, including biological process (BP), cell composition (CC), molecular function (MF), and KEGG pathways. The results were imported into bioinformatics software (http://www.bioinformatics.com.cn/). Sequentially, the highest pathway in the KEGG mapper (http://www.kegg.jp.org/) (Kanehisa and Sato, 2020) was highlighted to show its specific molecular mechanism in this pathway.

### Construction of the Drug–Compound-Target–Pathway-Disease (D-C-T-P-D) Network

Cytoscape (version 3.8.2) (Piñero et al., 2021) software was used to establish the D-C-T-P-D network model, and drugs, active compounds, cross genes. and diseases were introduced respectively. The top core pathway in the previous KEGG pathway enrichment analysis was inputted to establish the relationship between these elements and build a complete regulatory network by using the degree value for internal ranking.

### Molecular Docking

The Swiss dock (http://www.swissdock.ch/) (Grosdidier et al., 2011) database was used to analyze the molecular binding affinity of the molecular docking between the active components of CX and the core target for the treatment of OA. Target protein was obtained by comprehensively evaluating the resolution and release time in the Protein Data Bank (PDB) (www.rcsb.org) website (Carugo, 2021), and chemical structures of active components were downloaded from the PubChem database. Openbabel software was used to convert the SDF format into the mol2 format (O'Boyle et al., 2011). The specific binding sites and atomic distances between active components and proteins were determined by UCSF chimera software (Pettersen et al., 2004).

## Results

### Screened CX Compounds Targets and OA Disease Targets

**FIGURE 1:**
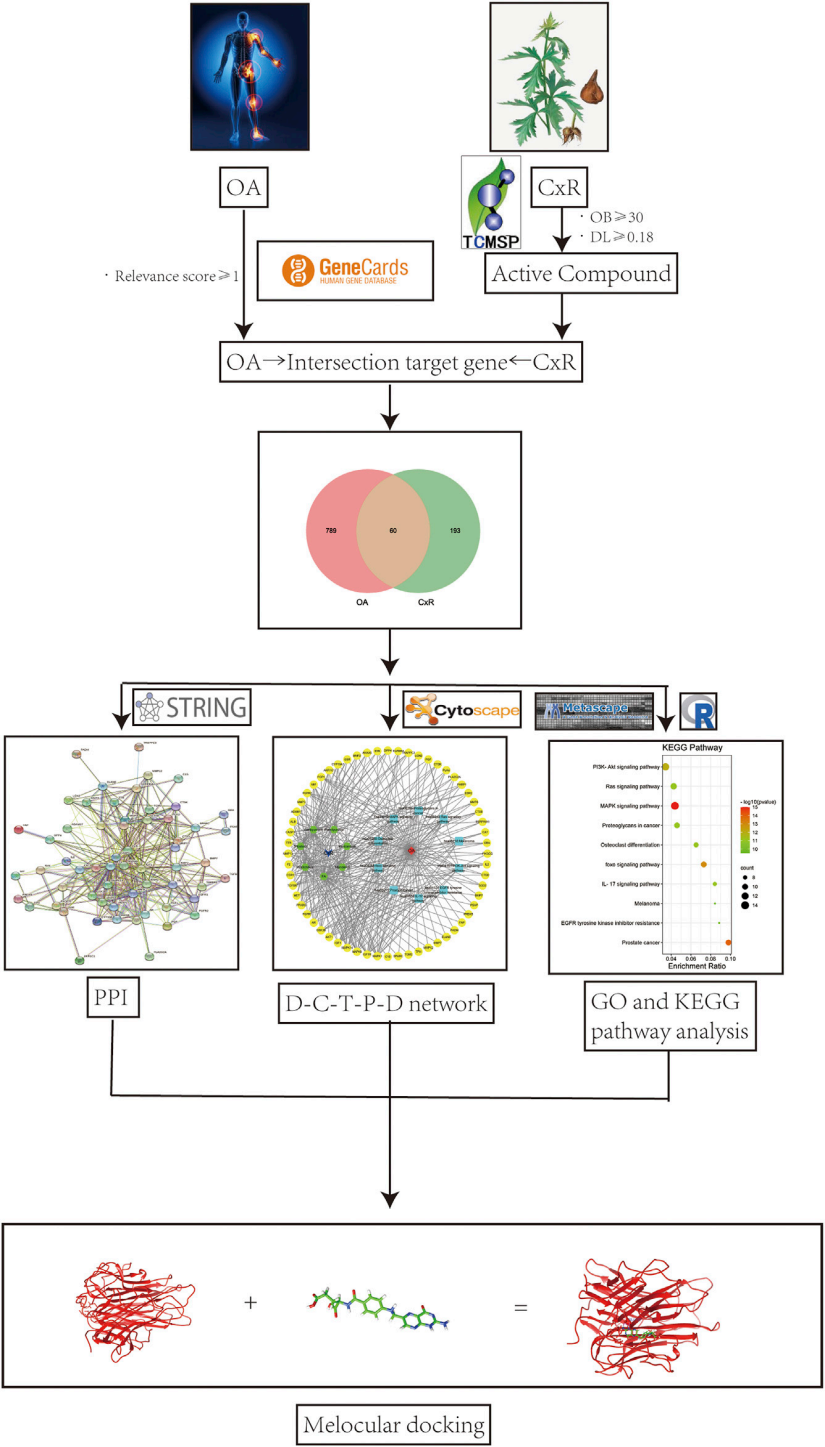
Flow chart of network pharmacology to study the potential molecular mechanism of CX in the treatment of OA.

**FIGURE 2:**
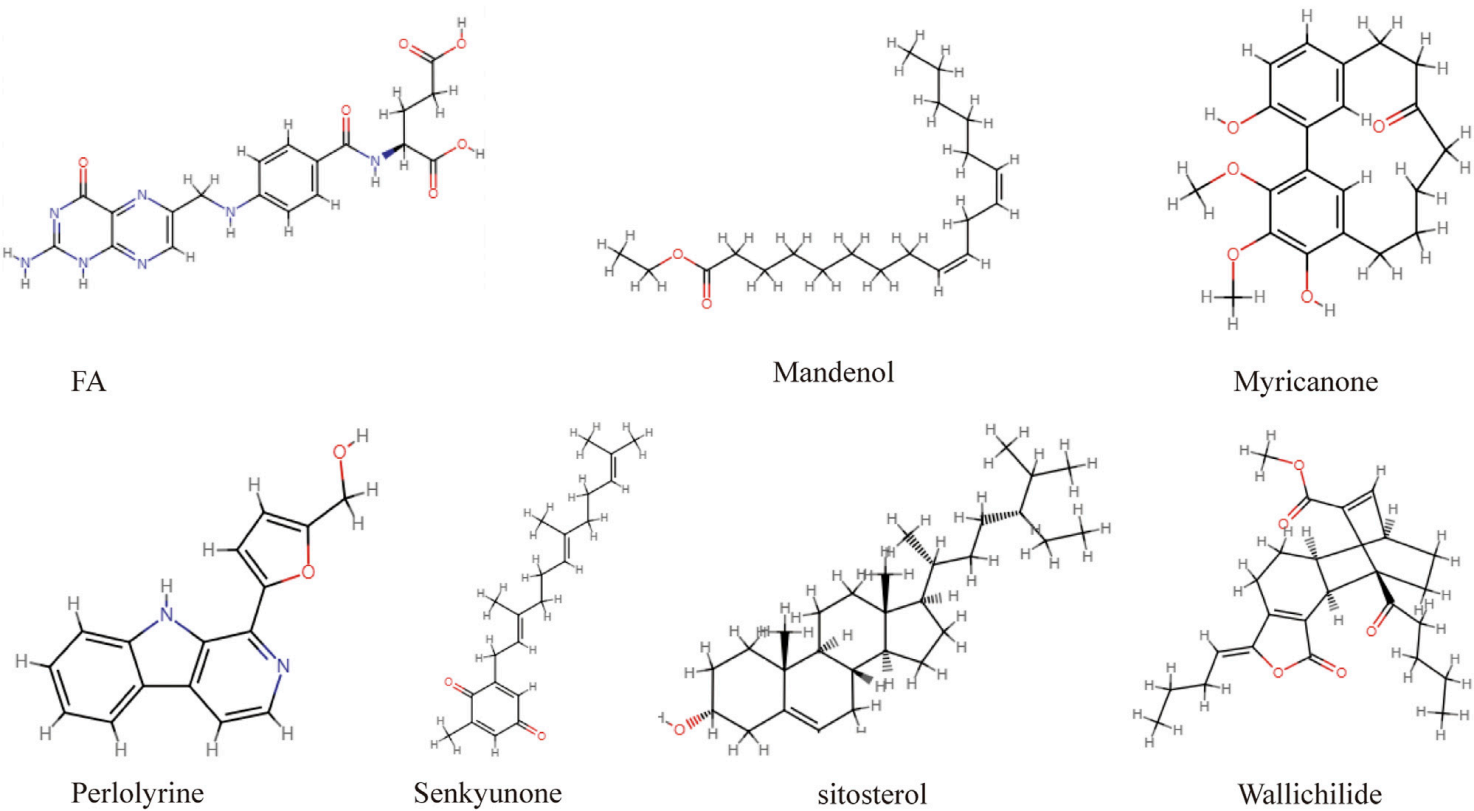
The chemical structure formula of active components of CX screened from the TCMSP database.

The flaw chart of this study as displayed in Figure 1. A total of 189 ingredients isolated from CX were collected from the TCMSP database. According to the screening criteria of OB ≥ 30% and DL ≥ 0.18, seven compounds were identified, including mandenol, myricanone, perlolyrine, senkyunone, wallichilide, sitosterol, and FA (structure in Figure 2, detail in Table 1). PharmMapper was used for target prediction, and the results were selected with norm fit >0.6 for subsequent analysis. We obtained 100 targets of mandenol, 56 of myricanone, 68 of perlolyrine, 116 of senkyunone, 152 of wallichilide, 98 of sitosterol, and 177 of FA. With repeat targets excluded, 253 drug targets were obtained finally. And a total 3,107 targets of osteoarthritis were identified from the GeneCards database. We selected the 849 results with reference score >1 for subsequent analysis. Both targets of CX and OA were imported into a Venn diagram and the 60 overlapped genes were the potential targets for CX anti-OA (entrez IDs shown in Table 2).

**TABLE 1:** The ADME characteristics of CX active compounds according to the TCMSP database.

NO.	Molecule ID	Molecule name	Molecular weight	OB(%)	DL
1	MOL001494	Mandenol (Ethyl linolate)	308.56	42	0.19
2	MOL002135	Myricanone	356.45	40.6	0.51
3	MOL002140	Perlolyrine	264.3	65.95	0.27
4	MOL002151	senkyunone	326.52	47.66	0.24
5	MOL002157	wallichilide	412.57	42.31	0.71
6	MOL000359	sitosterol	414.79	36.91	0.75
7	MOL000433	FA	441.45	68.96	0.71

OB, oral bioavailability; DL, drug-likeness.

**TABLE 2:** The detailed entrez IDs of 60 potential targets of CX anti-OA.

NO.	Target	Symbol	Entrez ID	NO.	Target	Symbol	Entrez ID
1	Disintegrin and metalloproteinase domain-containing protein 17	ADAM17	P78536	31	Neutrophil gelatinase-associated lipocalin	LCN2	P80188
2	Aldo-keto reductase family 1 member C1	AKR1C1	Q04828	32	Mitogen-activated protein kinase 1	MAPK1	P28482
3	RAC-alpha serine/threonine-protein kinase	AKT1	P31749	33	Mitogen-activated protein kinase 14	MAPK14	Q16539
4	Serum albumin	ALB	P02768	34	Mitogen-activated protein kinase 8	MAPK8	P45983
5	Annexin A5	ANXA5	P08758	35	Hepatocyte growth factor receptor	MET	P08581
6	Androgen receptor	AR	P10275	36	Macrophage migration inhibitory factor	MIF	P14174
7	Bone morphogenetic protein 2	BMP2	P12643	37	Macrophage metalloelastase	MMP12	P39900
8	Bone morphogenetic protein 7	BMP7	P18075	38	Collagenase 3	MMP13	P45452
9	Complement C1s subcomponent	C1S	P09871	39	Stromelysin-1	MMP3	P08254
10	Caspase-3	CASP3	P42574	40	Matrilysin	MMP7	P09237
11	Catalase	CAT	P04040	41	Neutrophil collagenase	MMP8	P22894
12	Cathepsin B	CTSB	P07858	42	Protein-arginine deiminase type-4	PADI4	Q9UM07
13	Cathepsin D	CTSD	P07339	43	Poly [ADP-ribose] polymerase 1	PARP1	P09874
14	Cathepsin K	CTSK	P43235	44	Placenta growth factor	PGF	P49763
15	Aromatase	CYP19A1	P11511	45	Phosphatidylinositol 4,5-bisphosphate 3-kinase catalytic subunit gamma isoform	PIK3CG	P48736
16	Dipeptidyl peptidase 4	DPP4	P27487	46	Phospholipase A2, membrane associated	PLA2G2A	P14555
17	Neutrophil elastase	ELANE	P08246	47	Urokinase-type plasminogen activator	PLAU	P00749
18	Estrogen receptor	ESR1	P03372	48	Peroxisome proliferator-activated receptor gamma	PPARG	P37231
19	Estrogen receptor beta	Esr2	Q92731	49	Peroxiredoxin-5, mitochondrial	PRDX5	P30044
20	Prothrombin	F2	P00734	50	Prosaposin	PSAP	P07602
21	Prolyl endopeptidase FAP	FAP	Q12884	51	Alpha-1-antitrypsin	SERPINA1	P01009
22	Fibroblast growth factor 1	FGF1	P05230	52	Superoxide dismutase [Mn], mitochondrial	SOD2	P04179
23	Fibroblast growth factor receptor 1	FGFR1	P11362	53	SPARC	SPARC	P09486
24	Fibroblast growth factor receptor 2	FGFR2	P21802	54	Tyrosine-protein kinase SYK	SYK	P43405
25	Lysosomal acid glucosylceramidase	GBA	P04062	55	TGF-beta receptor type-1	TGFBR1	P36897
26	Glycogen synthase kinase-3 beta	GSK3B	P49841	56	TGF-beta receptor type-2	TGFBR2	P37173
27	Glutathione reductase, mitochondrial	GSR	P00390	57	Protein-glutamine gamma-glutamyltransferase 2	TGM2	P21980
28	Insulin-like growth factor I	IGF1	P05019	58	Triosephosphate isomerase	TPI1	P60174
29	Insulin-like growth factor 1 receptor	IGF1R	P08069	59	Trafficking protein particle complex subunit 3	TRAPPC3	O43617
30	Interleukin-2	IL-2	P60568	60	Transthyretin	TTR	P02766

### PPI Network Analysis

**FIGURE 3:**
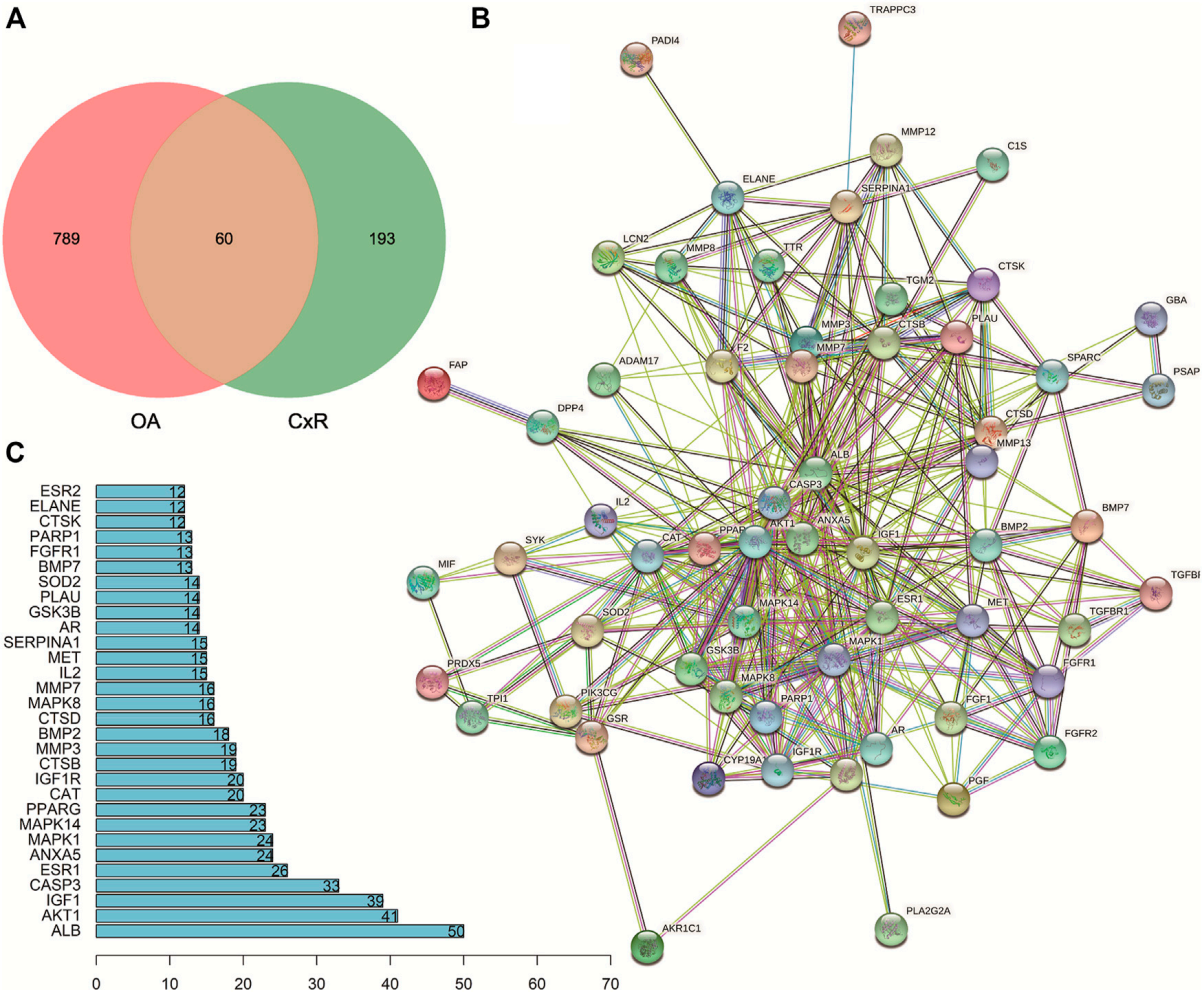
Potential targets of CX anti-OA and the PPI network. **(A)** Venn diagram of potential targets. **(B)** The PPI network of 60 targets according to the STRING database. **(C)** Top 30 targets ranked by the degree value.

**FIGURE 4:**
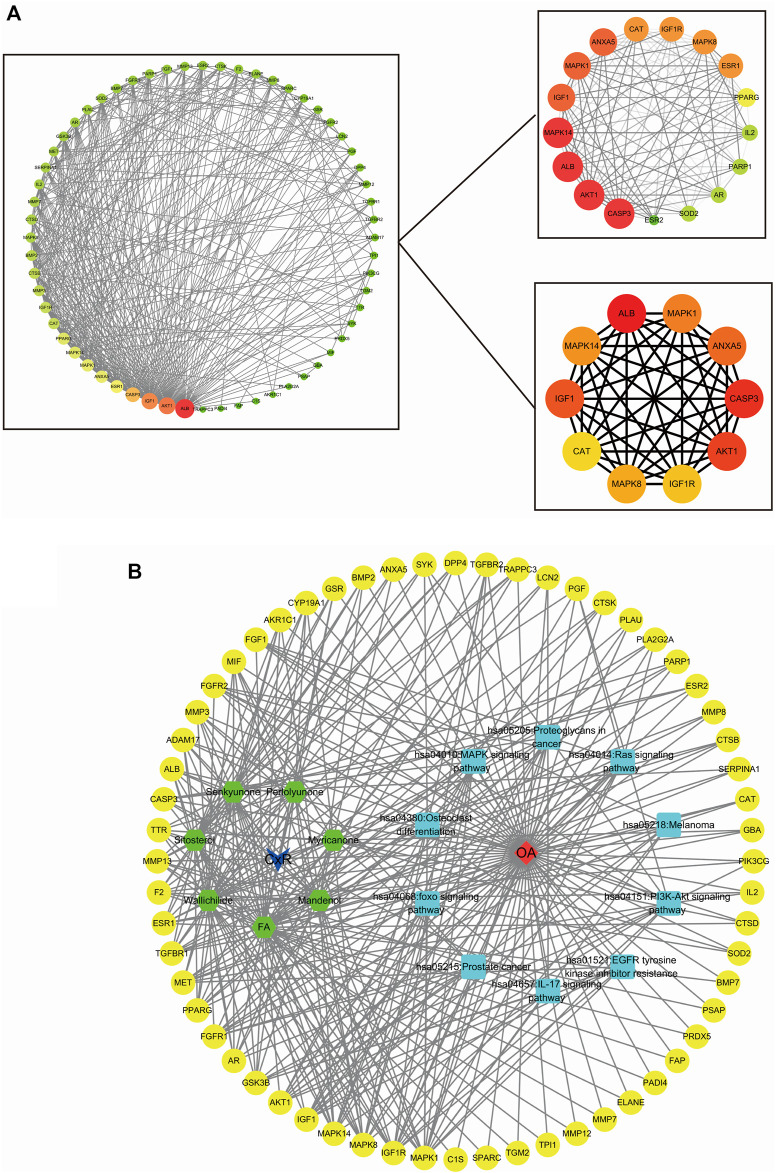
Plug-in of Cytoscape was used to select the molecular complexes and core targets and the D-C-T-P-D network **(A)** MCODE and CytoHubba plug-ins of the Cytoscape software were used to choose the molecular complexes and core targets, respectively. **(B)** The drug-compound-target-pathway-disease network showed the potential mechanism of CX to treat OA.

A total of 60 predicted targets were imported into STRING for PPI network analysis (Figure 3). The network complex included 60 nodes and 394 edges. The Cytoscape software was used to visualize and analyze the network by calculating centrality and other parameters. All the targets were arranged into circles according to these parameters. The high centrality value represented the important role in the network. Then the plug-ins MCODE and CytoHubba selected the core targets (Figure 4A). The top 10 core targets were ALB, AKT1, IGF1, CASP3, MAPK1, ANXA5, MAPK14, CAT, and IGF1R, respectively.

### GO Function and KEGG Pathway Enrichment Analysis

**FIGURE 5:**
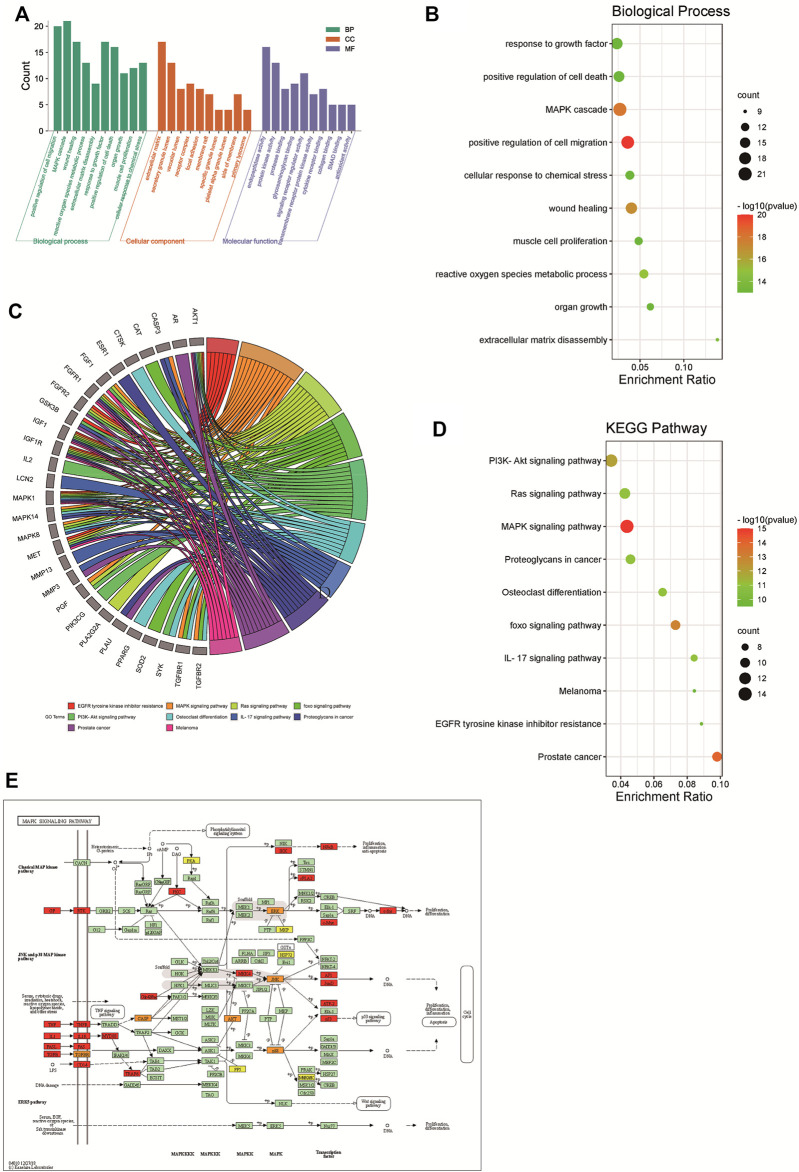
GO function and KEGG pathway enrichment analyses of CX in the treatment of OA. **(A)** The GO function analysis, including biological process (BP), cellular component (CC), and molecular function (MF). **(B)** Bubble diagram of BP enrichment. **(C)** Gene ontology chord of the top 10 pathways in the CX anti-OA **(D)** Bubble diagram of KEGG pathway enrichment. **(E)** The MAPK pathway was colored by the KEGG mapper, the orange targets represent CX anti-OA, yellow represents other targets of CX not involved in OA treatment, and red represents other targets of OA in the MAPK pathway.

A total of 970 GO items were obtained from the Metascape database (*p* < 0.01), included 871 BP items, 40 CC items, and 59 MF items. We further selected the top 10 BP, CC, and MF catalogs for visualization (Figure 5A). In the histogram, the ordinary axis represents the degree of enrichment. According to our results of BP (Figure 5B), the function of active components of CX in osteoarthritis mainly focused on positive regulation of cell migration, MAPK cascade, wound healing, reactive oxygen species metabolic process, extracellular matrix disassembly, response to growth factor, positive regulation of cell death, organ growth, muscle cell proliferation, and cellular response to chemical stress. The MF items mainly included endopeptidase activity, protein kinase activity, protein binding, glycosaminoglycan binding, signaling receptor regulator activity, transmembrane receptor protein kinase activity, cytokine receptor binding, collagen binding, SMAD binding, and antioxidant activity. The abundant GO functions can also contribute to explaining to a certain extent that CX can be used to treat osteoarthritis and other diseases. KEGG pathway enrichment analysis showed that CX was mainly involved in 191 signaling pathways (*p* < 0.01) (Figures 5C,D), the top 10 enriched pathways were visualized by a bubble chart, the degree of gene enrichment was represented by abscissa, the amount of gene enrichment was represented by the bubble size, and *p* value was represented by the color depth. The main pathways of enrichment included EGFR tyrosine kinase inhibitor resistance, the MAPK signaling pathway, Ras signaling pathway, FoxO signaling pathway, PI3K-Akt signaling pathway, osteoclast differentiation, IL-17 signaling pathway, proteoglycans in cancer, prostate cancer, and melanoma. The KEGG mapper was used to color in targets in the MAPK pathway, the targets of CX anti-OA were colored in orange, other targets of CX but not involved in OA were colored in yellow, and other targets of OA in the MAPK pathway were colored in red (Figure 5E).

### D-C-T-P-D Network Analysis

The D-C-T-P-D network of CX for the treatment of osteoarthritis is displayed in Figure 4B, showing the complex relationship between CX and osteoarthritis, including 79 total nodes (7 compound nodes, 60 target nodes, 10 core pathways, one CX node, and one OA node) and 359 edges. The targets, drug, and OA were represented by yellow circles, a blue arrow, and a red prism, respectively. While, the active compounds and core pathways were shown as green hexagons and blue rectangles, respectively. In the compound section, the degree of FA, perlolyunone, sitosterol, senkyunone K, wallichilide, mandenol, and myricanone was 42, 18, 23, 30, 37, 25 and 15, respectively, indicating that FA may be the most important active component in the treatment of osteoarthritis. Similarly, the degree of the MAPK pathway was 15, which was the most important signaling pathway, and that of MAPK1 was 16, which was the most important potential target predicted.

### Molecular Docking Findings

**FIGURE 6:**
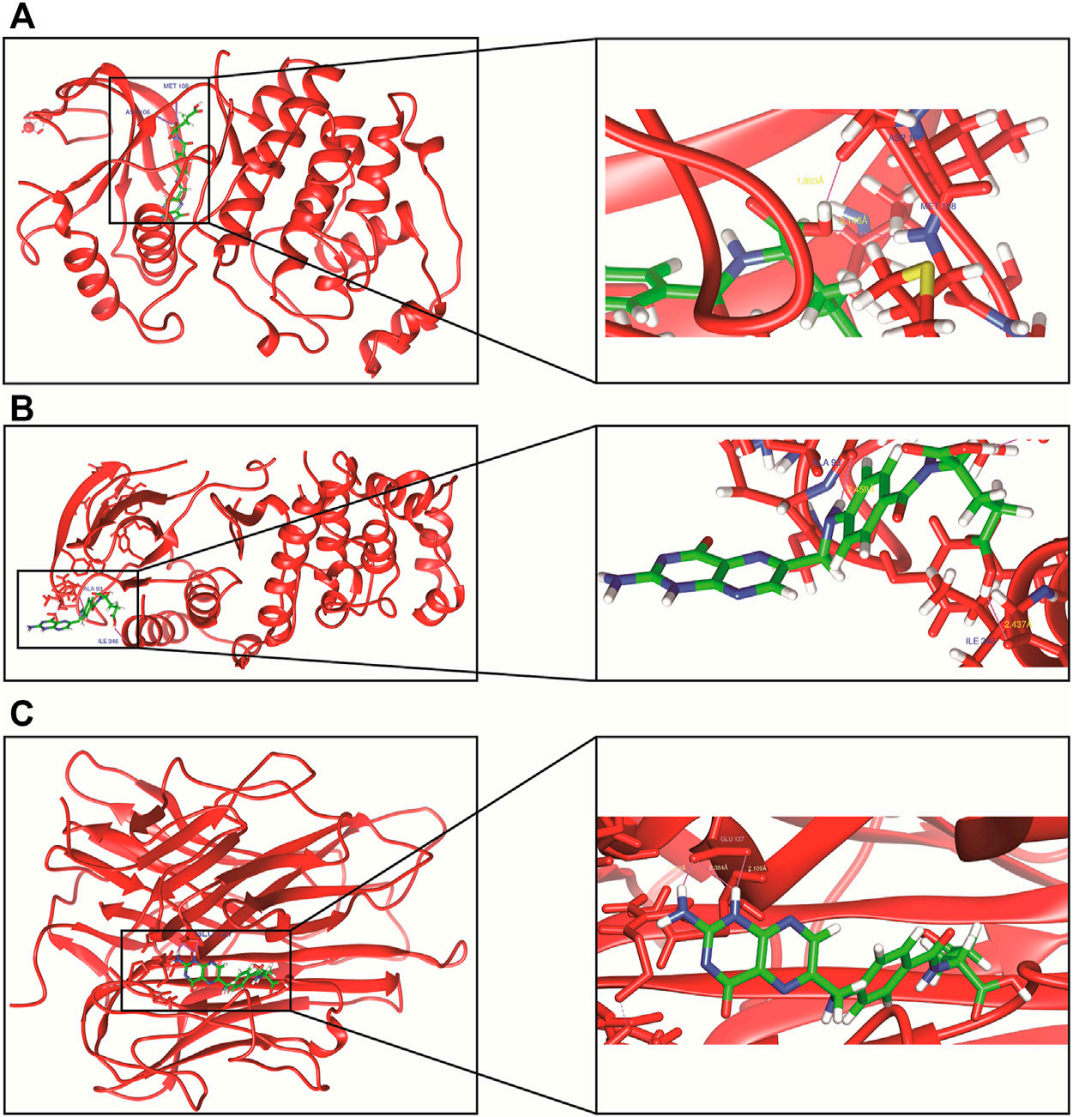
The molecular docking of FA and core targets. **(A)** FA docking with MAPK1. **(B)** FA docking with MAPK14. **(C)** FA docking with TNF-α.

Finally, we employed molecular docking to determine the possibility of binding between the core target and the CX active compounds via applying the Swiss dock website (Figure 6). The previous literature proved that a binding affinity of < -4.25 kcal/mol indicated that the two molecules had a standard binding ability, < -5.0 kcal/mol meant good binding, while < -7.0 kcal/mol suggested strong binding activity (Saikia and Bordoloi, 2019). In our study, we docked two top targets in the MAPK pathway (MAPK1 and MAPK14) and TNF-α with seven active monomers of CX respectively. The results illustrated that most of results were < - 7 kcal/mol, and the binding energy between FA and MAPK1 as well as MAPK14 was < - 9 kcal/mol (Table 3). The docking results of FA and three targets were refined by exploring the specific binding sites and the spatial distance in UCFS chimera. The result showed that in MAPK1 (PDB ID:7AUV), FA had hydrogen bonds with amino acid residues of ASP-106 and MET-108, the distance was 1.803 Å and 2.145 Å, respectively. In MAPK14 (PDB ID:6SP9), FA and amino acid residues ALA-93 (2.458 Å) and ILE-346 (2.437 Å) had hydrogen bonds. In TNF-α (PDB ID:1A8M), FA had three hydrogen bonds with amino acid residues of GLU-127 (2.029 Å, 2.384 Å, and 2.109 Å).

**TABLE 3:** The detailed molecular docking messages of seven CX compounds with MAPK1, MAPK14, and TNF-α.

	FA	Mandenol	Myricanone	Perlolyunone	Senkyunone	Sitosterol	Wallichilide
MAPK1	−9.24	−7.96	−6.98	−7.39	−7.82	−7.97	−7.93
MAPK14	−9.4	−8.94	−7.39	−8.07	−8.47	−7.42	−8.13
TNF-a	−8.51	−7.74	−6.69	−7.24	−7.12	−7.28	−7.26

## Discussion

Osteoarthritis is a common degenerative joint disease and the incidence rate of middle-aged and elderly people is increasing every year, which negatively impacts the health and quality of sufferers’ lives (Martel-Pelletier et al., 2016). Current treatment of osteoarthritis mainly focuses on relieving the pain and other discomfort symptoms of patients, which does little to stop or slow down the progression of the disease. The currently known functions of TCM are tonifying the liver and kidney, relaxing tendons and activating blood circulation, and dredging collateral and relieving pain (Xu et al., 2017). As an important blood-activating and detumescence drug, Ligusticum chuanxiong has a long history in the treatment of osteoarthritis, but the exploration of its specific molecular mechanism is far from sufficient (Luo Y. et al., 2020). There have been a few studies on osteoarthritis treatment by CX, most of them focused on the isolated compounds from CX and lacked systematic insights and depth (Zhou et al., 2019a). As we all know, many ingredients of Chinese herbal medicine have certain curative effects in experiments, but they have not been used in the clinic. The reason for the phenomenon is that those studies ignored the physicochemical property of CX as a drug (Anand et al., 2007). For example, many compounds have poor water solubility and oral availability, so it is difficult to effectively achieve the concentration required for treatment (Kunnumakkara et al., 2017).

With the emergence of modern bioinformatics, people are beginning to pay more attention to the combination of computer science and biology. Data mining can help us find valuable information from big data and guide us to carry out meaningful research (Gauthier et al., 2019). Based on a large number of experimental data and clinical trial results, network pharmacology could be used to study the potential mechanism of clinical treatment and prevention of diseases, and is especially suitable for the complex components and multi-target and multi mechanism features of TCM (Zhang et al., 2019). Therefore, this study was the first to systematically reveal the mechanism of CX anti-OA. In addition, the molecular docking model between the compound and the target also showed a good combination, which was further verified by the internal relationship between CX and OA.

Through the D-C-T-P-D network, it was determined that FA, wallichilide, and senkyunone were the most important potential components of CX anti-OA. Previous studies have indicated that the folate receptor is overexpressed on activated macrophages, which are closely related to osteoarthritis (Tsuneyoshi et al., 2012). Some literature has also showed that folic acid deficiency (FD) can mediate synovial cell apoptosis by affecting the excessive production of reactive oxygen species (ROS) induced by mitochondrial complex II and NOX and the sharp release of intracellular calcium (Ca2 +) concentration (Hsu et al., 2016). Besides, Duan W et al. proved that a folate receptor-targeted nanocarrier system can effectively block the NF-κB signaling pathway and reduce the expression of proinflammatory cytokines, so as to significantly inhibit the progression of arthritis in a mouse model (Duan and Li, 2018). Gelmini F et al. conducted a small clinical trial and found that ointment containing sitosterol used to treat five patients with osteoarthropathy (three times a day for three consecutive weeks) reduced the inflammatory characteristics of hands and knees in all patients without obvious adverse reactions (Gelmini et al., 2016). Cheng BC et al. pointed out that sitosterol can target multiple different genes in the NF-κB signaling pathway, such as MMP and COX-2, to reduce inflammatory cytokines and chemokine TNF-α, IL-1 β, IL-6, and CCL5, thereby decreasing oxidative stress and inhibiting inflammation (Cheng et al., 2016). Besides, Shivnath N et al. found that sitosterol can bind to the chondrocyte membrane and inhibit the NF-κB pathway to downregulate the expression of MMP and inhibit the catabolism of the cartilage matrix (Shivnath et al., 2021). In general, the seven active components of CX have different degrees of therapeutic effects on OA, involving numerous inflammatory cells and signaling pathways.

Then we applied a PPI network. The results showed that there were abundant interactions among targets, which made it easier to produce cascade effects. The core targets were ALB, AKT1, IGF1, CASP3, MAPK1, ANXA5, MAPK14, CAT, and IGF1R. These targets corresponded to multiple components in CX; this point fully reflected the characteristics of multi-target and multi-component traditional Chinese medicine. Some studies have shown that the osteophyte formation of OA in AKT1-knockout mice was blocked in the animal model of osteoarthritis. Further studies have found that the mechanism was the inhibition of the expression of calcification inhibitor nucleotide pyrophosphatase/phosphodiesterase 1 (Fukai et al., 2010). And IGFs, including IGF1 and IGF1R, played an important role in chondrocyte growth and chondrogenesis (Claessen et al., 2012), while CASP3 was closely related to chondrocyte apoptosis (Wang et al., 2019). Deng Z et al. found that MAPK14 was highly expressed in patients with osteoarthritis as the death promoter of chondrocytes (Deng et al., 2021), while the overexpression of MAPK1 could reduce the inflammatory injury of chondrocytes, which was induced by IL-1 β (Hu et al., 2018). Annexin A5 (ANXA5) played an important role in matrix vesicle-mediated biomineralization during endochondral ossification and osteoarthritis (Grskovic et al., 2012). CAT (catalase) could clear ROS in chondrocytes and inhibit apoptosis induced by TNF-α, and then regulate the growth of chondrocytes by the death signaling pathway (van Gastel et al., 2020).

We discovered that the “PI3K-Akt signal pathway”, “MAPK signal pathway”, “osteological differentiation”, and “IL-17 signal pathway” were the potential key mechanisms of CX in the treatment of OA. In our study, MAPK1 and MAPK14 may be involved in the main mechanism of CX in the treatment of OA, as the most potential core molecules in the MAPK pathway. At the same time, the molecular docking results also proved that the active components of CX were well docked with MAPK1 and MAPK14. A variety of stimulating factors or toxic injury can activate the PI3K-Akt signaling pathway, and then regulate basic cell functions such as transcription, proliferation, and survival (Xue et al., 2017). ERK (MAPK1) and p38 (MAPK14) are the important ways for eukaryotes to regulate cell structure and function, and their activations were closely related to cartilage injury in OA. MMP, which is regulated by ERK and p38, is closely related to chondrocyte proliferation, apoptosis, and differentiation (Chen Y. et al., 2018). And the IL-17 signaling pathway played an important role in acute and chronic inflammatory and immune regulation (Liu et al., 2019). Osteoclasts are the only cells responsible for bone resorption in the body and play an important role in the process of OA. So, regulating osteoclast differentiation is a significant method to treat OA (Wang S. et al., 2020). Other pathways which we have been enriched, such as the TNF pathway and NF-κB pathway were also classic pathways highly related to OA.

In conclusion, our study used network pharmacology to elaborate the potential mechanism of CX in the treatment of osteoarthritis, and intuitively verified its effectiveness through molecular docking. Our study showed that CX can act on chondrocytes, synovial cells, and other cells closely related to the occurrence and development of OA. Meanwhile, CX was effective in the treatment of osteoarthritis by multi-target and multi-channel properties. However, there are still some deficiencies in this study, and the reliability of these conclusions needs further basic research to verify. Our research provided a new direction for the further research of CX in the treatment of osteoarthritis.

## Conclusion

Our research, based on bioinformatics, found that Ligusticum chuanxiong was effective in the treatment of osteoarthritis with the characteristics of multi-component, multi-target, and multi-pathway properties. Our results also guide directions for subsequent basic experiments.

## Data Availability

The original contributions presented in the study are included in the article/Supplementary Material, further inquiries can be directed to the corresponding authors.
